# DReLAB - Deep REinforcement Learning Adversarial Botnet: A benchmark dataset for adversarial attacks against botnet Intrusion Detection Systems

**DOI:** 10.1016/j.dib.2020.106631

**Published:** 2020-12-08

**Authors:** Andrea Venturi, Giovanni Apruzzese, Mauro Andreolini, Michele Colajanni, Mirco Marchetti

**Affiliations:** a Department of Engineering “Enzo Ferrari”, University of Modena and Reggio Emilia, Italy; b Hilti Chair of Data and Application Security, University of Liechtenstein, Vaduz, Liechtenstein; c Department of Physics, Computer Science and Mathematics, University of Modena and Reggio Emilia, Italy; d Department of Informatics, Science and Engineering, University of Bologna, Italy

**Keywords:** Cyber security, Adversarial attacks, Deep reinforcement learning, Intrusion detection system, Botnet

## Abstract

We present the first dataset that aims to serve as a benchmark to validate the resilience of botnet detectors against adversarial attacks. This dataset includes realistic adversarial samples that are generated by leveraging two widely used Deep Reinforcement Learning (DRL) techniques. These adversarial samples are proved to evade state of the art detectors based on Machine- and Deep-Learning algorithms. The initial corpus of malicious samples consists of network flows belonging to different botnet families presented in three public datasets containing real enterprise network traffic. We use these datasets to devise detectors capable of achieving state-of-the-art performance. We then train two DRL agents, based on *Double Deep Q-Network* and *Deep Sarsa*, to generate realistic adversarial samples: the goal is achieving misclassifications by performing small modifications to the initial malicious samples. These alterations involve the features that can be more realistically altered by an expert attacker, and do not compromise the underlying malicious logic of the original samples. Our dataset represents an important contribution to the cybersecurity research community as it is the first including thousands of automatically generated adversarial samples that are able to thwart state of the art classifiers with a high evasion rate. The adversarial samples are grouped by malware variant and provided in a CSV file format. Researchers can validate their defensive proposals by testing their detectors against the adversarial samples of the proposed dataset. Moreover, the analysis of these samples can pave the way to a deeper comprehension of adversarial attacks and to some sort of explainability of machine learning defensive algorithms. They can also support the definition of novel effective defensive techniques.

## Specifications Table

**Table:** 

Subject	Computer Science Applications
Specific subject area	Cyber security, Adversarial attacks, Deep Reinforcement Learning, Intrusion Detection System, Botnet
Type of data	CSV files
How data were acquired	We submit malicious botnet samples from three public datasets to DRL agents trained to evade botnet detectors by inserting tiny and feasible feature modifications. The dataset is composed of the modified samples that were able to evade the detection provided by the botnet detectors.
Data format	Raw: Adversarial samples, generated by leveraging DRL agents based on Double Deep Q-Network and Deep Sarsa, that evade the detection of botnet detectors based on Random Forest and Wide and Deep [1] classifiers. As additional contributions, we also provide: the original malicious samples used for the generation of the adversarial samples; the benign traffic to ease the reproduction of our experiments; the traffic of the BOTNET2014 dataset in a labelled network flow format.
Parameters for data collection	In the following sections, we provide the most important parameters for the Random Forest and Wide and Deep classifiers in Table 8 and Table 9; while in Table 11 we report the most important settings for the DRL agents.
Description of data collection	The collection of data is divided in three phases: DATA NORMALIZATION: We retrieve three public datasets containing a mix of legitimate and botnet traffic captured in enterprise networks. PREPROCESSING: The obtained network flows are then subject to a preprocessing step with a twofold goal: (i) removing some outliers and (ii) obtaining a uniform feature set. ADVERSARIAL SAMPLE GENERATION: The resulting malicious flows are used to devise the botnet detectors used as “target” for the DRL agents. Then, we train DRL agents to achieve misclassifications by performing small alterations to some netflow features. The adversarial samples that have obtained a misclassification form the DReLAB dataset.
Data source location	Institution: Department of Engineering “Enzo Ferrari”, University of Modena and Reggio Emilia City: Modena Country: Italy Primary data sources: CTU13 [2]:https://www.stratosphereips.org/datasets-ctu13#CSE-CIC-IDS2018[3]:https://registry.opendata.aws/cse-cic-ids2018/BOTNET2014[4]:https://www.unb.ca/cic/datasets/botnet.html For simplicity, in the rest of the paper we will refer to the above-mentioned datasets respectively as CTU, CICIDS and BOTNET.
Data accessibility	Repository name: DReLAB dataset – Deep Reinforcement Learning Adversarial Botnet dataset [5] Data identification number:10.17632/nf22d786tj.1 Direct URL to data: https://data.mendeley.com/datasets/nf22d786tj/1 Github tutorial: https://github.com/andreaventuri01/DReLAB_tutorial
Related research article	Apruzzese, Giovanni and Andreolini, Mauro and Marchetti, Mirco and Venturi, Andrea and Colajanni, Michele. “Deep Reinforcement Adversarial Learning against Botnet Evasion Attacks.” *IEEE Transactions on Network and Service Management*. DOI: 10.1109/TNSM.2020.3031843[6]

## Value of the Data

• Our dataset includes realistic adversarial samples automatically generated by leveraging DRL techniques against botnet detectors. The dataset aims to serve as a benchmark for evaluating the robustness of novel ML-based IDS, avoiding the researchers the manual production of adversarial samples to test their solutions.
• Researchers can consider this dataset for several reasons: it can be used to validate the efficacy of existing countermeasures against adversarial threats; moreover, it can help cybersecurity researchers to propose novel methods to counter adversarial attacks against botnet detectors.
• Our dataset al1lows researchers to focus only on the proposal of novel defensive strategies without working on procedures for generating meaningful adversarial samples.
• Furthermore, the proposed dataset includes samples from a novel source, CSE-CIC-IDS2018 [3], that is not considered in the main research paper [6]. This additional source further enhances the quality of the proposed dataset as it contains thousands of adversarial samples belonging to several botnet families and resembling multiple attack scenarios.

## 1 Data Description

**Fig. 1:**
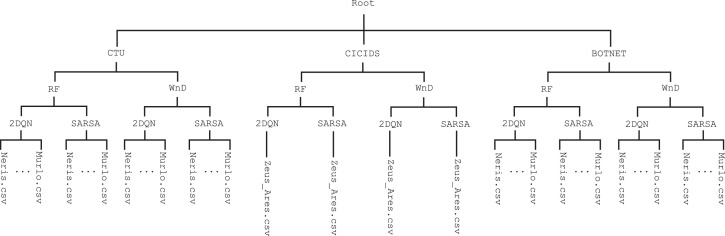
Dataset structure.

We organize the structure of the dataset as shown in Fig. 1. From top to bottom, we divide the dataset into three directories corresponding to the original datasets CTU, CICIDS and BOTNET, respectively. In each of these dataset-directories, we separate the adversarial samples according to the ML algorithm at the basis of the “target” botnet detector that they have evaded: **Random Forest** (**RF**), and **Wide and Deep** (**WnD**). We then distinguish the DRL algorithm that has modified the samples: **Double Deep Q-network** (**2DQN**) and **Deep Sarsa** (**Sarsa**). Finally, each CSV file contains the adversarial samples belonging to a specific botnet family of the original datasets. As an example, the file CTU/RF/2DQN/Neris.csv contains the adversarial samples of the Neris botnet family from the CTU dataset that have been modified by the **2DQN** agent to evade the **RF**-based botnet classifier, trained to detect the samples of Neris. In Table 1 we summarize the number of samples contained in each CSV file of the dataset. Each entry in the table contains the amount of adversarial samples that evaded the botnet classifier for each botnet family of the three datasets using the **2DQN** and **Sarsa** agents. As an example, CTU/RF/2DQN/Neris.csv contains 58,429 samples. Similarly, CICIDS/WnD/Sarsa/Zeus_Ares.csv contains 286,160 samples.

**Table 1:** Number of adversarial samples in each CSV file.

Dataset		CTU					CICIDS	BOTNET				
Detector	DRL agent	Neris	Rbot	Virut	Menti	Murlo	Zeus&Ares	Neris	Rbot	Virut	Menti	Murlo
**RF**	**2DQN**	58,429	27,452	31,941	2825	1106	285,351	64,082	29,503	564	7754	5815
	**Sarsa**	58,213	27,425	31,317	2823	1106	285,905	65,551	28,267	548	7920	4982
**WnD**	**2DQN**	54,729	21,709	15,146	2825	1106	285,282	67,469	29,599	635	7893	5812
	**Sarsa**	53,920	26,320	11,001	2824	1106	285,905	66,547	29,515	634	7923	4212

We report in Table 2 the set of features associated to each sample included in the dataset. As data come from different sources, their relative feature sets differ accordingly to the available features in the original dataset. For example, CICIDS does not provide information on the *Source* and *Destination IP Addresses* of the flows as CTU and BOTNET. Similarly, the CTU flows contain the *Type of Service* feature, while the other datasets do not give this information. We also add in a dedicated feature the number of actions that each sample required to evade the detection in a dedicated feature.

**Table 2:** Feature set of our samples.

Feature	Description	Type
IP Src/Dst Type*	It indicates whether the *Source* / *Destination Ip Address* belongs to the internal network or not.	Binary (0 external, 1 internal).
SrcPortWellKnown* / DstPortWellKnown	1 if the *Source* / *Destination Port* number is between 0 and 1023. 0 otherwise.	Binary (0 or 1).
SrcPortRegistered* / DstPortRegistered	1 if the *Source* / *Destination Port* number is between 1024 and 49,151. 0 otherwise.	Binary (0 or 1).
SrcPortPrivate* / DstPortPrivate	1 if the *Source* / *Destination Port* number is higher than 49,151. 0 otherwise.	Binary (0 or 1).
*Duration*	Duration in seconds of the flow communication.	Float
*InBytes*	Incoming Bytes.	Float
*OutBytes*	Outgoing Bytes.	Float
*TotBytes*	Total number of bytes in the communication. Obtained by summing *OutBytes* and *InBytes* feature values.	Float
*TotPkts*	Total number of packets in the communication.	Float
*BytesPerPkt*	Average number of bytes exchanged per packet. Ratio between *TotBytes* and *TotPkts*.	Float
*PktsPerSec*	Average number of packets exchanged per second. Ratio between *TotPkts* and *Duration*.	Float
*RatioOutIn*	Ratio between *OutBytes* and *InBytes*.	Float
State*	State of the TCP communication.	Categorical
Direction*	Direction of the communication.	Categorical
*Protocol*	Type of protocol considered in the flow. (Only TCP)	Categorical
Src/Dst ToS**	Source and Destination Type of Service.	Categorical
*Number of Actions*	Number of actions required to obtain an evasion	Integer

A * means that the feature is not present in flows coming from the CICIDS dataset. A ** means that the feature is not present in flows coming from both CICIDS and BOTNET datasets.

Along with our adversarial samples, we also provide the following files:

• BOTNET/raw_labelled_flow.csv, which contains the already labeled flow version of the BOTNET PCAP traffic obtained following the process indicated in the next section (see Data Normalization phase). BOTNET/argus.conf and BOTNET/ra.conf are the configuration files we use for this procedure.
• <dataset>/malicious/ is a directory for each of the considered dataset which contains the malicious samples obtained at the end of the preprocessing phase. These are the original malicious samples we exploit to devise the adversarial ones.
• <dataset>/benign.csv contains the benign flows from each dataset at the end of the preprocessing phase.

## 2 Experimental Design, Materials and Methods

We consider the malicious flows provided by three publicly available datasets for botnet detection (CTU, CICIDS and BOTNET) as a basis for crafting realistic adversarial samples. The malicious flows are submitted to Deep Reinforcement Learning (DRL) agents that are trained to automatically modify the samples and to generate their evading “adversarial” versions.

The entire procedure consists of three phases: *Data Normalization, Preprocessing*, and *Adversarial Sample Generation*.

### 2.1 Data normalization

This phase aims to obtain a common representation of the data provided by the three considered datasets:

• CTU-13 [2] is a dataset that is captured at the Czech Technical University in Prague. It collects labeled network traffic in 13 collections (called scenarios): in each scenario, the authors executed a specific botnet variant and recorded its malicious activity along with normal and background traffic in PCAP files. Then, these files were converted in network flows through Argus^1^ that is a network audit system generating flows from raw network packets data. A network flow (netflow) can be considered as a tabular representation of the network traffic, in which the information of the connection between two hosts in the network is gathered in a single entry. Table 3 reports the features of the network flows of the CTU dataset. Before the preprocessing phase, we merge the malicious flow belonging to each botnet family in dedicated collections. Thus, we obtain five collections containing only malicious flows belonging to five different botnet variants (Neris, Rbot, Virut, Murlo, Menti). Moreover, we create a separate collection containing all the benign flows included in the CTU. As the CTU dataset contains a limited amount of malicious samples belonging to Sogou and NSIS.ay botnet, we do not consider these two variants.

**Table 3:** Feature set for the CTU dataset.

**Feature**
*Start Time*
*Source/Destination IP Address*
*Source/Destination Port*
*Protocol*
*Duration*
*Direction*
*State*
*Source/Destination Type of Service*
*Total Packets*
*Total Bytes*
*Source Bytes*
*Label*
• CSE-CIC-IDS2018 [3] is a dataset coming from a collaborative project between the Communications Security Establishment (CSE) and the Canadian Institute for Cybersecurity (CIC). The aim was to generate a dataset for intrusion detection referring to multiple attack scenarios. The dataset is publicly available on Amazon AWS.^2^ It includes thousands of labeled netflows with 80 features extracted through CICFlowMeter that is a network traffic flow generator from the CIC Institute. For space reasons, we do not list all the 80 features of the dataset, instead we report in Table 4 the features we will analyze in the next phases. Since we focus on botnet traffic, we consider only the flows collected on Friday 02–03–2018, that includes data related to the use of Zeus and Ares botnet variants. As we cannot distinguish the flows belonging to each botnet, we create one collection for malicious samples referring to both Zeus and Ares botnets, and one collection with the benign flows.

**Table 4:** Main features for the CICIDS dataset.

**Feature**
*Destination Port*
*Protocol*
*Duration*
*Source/Destination Bytes*
*Bytes per Packet*
*Bytes per Second*
*Total Packets*
*Total Bytes*
*Packets per Second*
*Ratio Out/In*
*Label*
• BOTNET2014 [4] is a dataset provided by the Canadian Institute for Cybersecurity. The authors used an overlay methodology to merge three different data traces: ISOT [7], ISCX 2012 IDS [8], and botnet traffic generated by the Malware Capture Facility Project. Unlike the other considered datasets, the authors provide data as full packet captures (PCAP) format: ISCX_Botnet_Training.pcap and ISCX_Botnet_Testing.pcap. In order to convert PCAP in network flows and obtain the final CSV files, we truncate these files to protect the user privacy (as indicated by the authors of the CTU dataset^3^), and then we uses argus and ra tools. We facilitate the reproducibility of our experiments by providing:
  ○ the configuration files: argus.conf and ra.conf (see the previous section),
  ○ and the adopted commands:
    i argus -r InputFile.pcap -F argus.conf > OutFile.argus
    ii ra -r InputFile.argus -F ra.conf > OutFile.csv
 At the end of these operations we obtain two CSV files, respectively from the training and testing original PCAP files. As a similar division is useless for the purposes of our dataset, we merge these files, and generate one file containing the entire BOTNET traffic in netflow format. As indicated by the authors of the dataset, we label as malicious the flows containing the malicious IP addresses listed in the Web page of the dataset^4^ either in their Source or Destination IP features, and we label as benign the remaining flows. In this way, we obtain a labeled netflow representation of the traffic of the BOTNET dataset. As a further contribution, we provide the labeled network flows that is obtained after the above operations. In Table 5 we report the feature set for this dataset. At the end, we divide the flows pertaining to each botnet family by considering the botnet variant contained a sufficient amount of flows: Neris, Rbot, Virut, Murlo, Menti. The benign flows are gathered in a dedicated collection.

**Table 5:** Feature set for BOTNET dataset.

**Feature**
*Start Time*
*Source/Destination IP Address*
*Source/Destination Port*
*Protocol*
*Duration*
*Direction*
*State*
*Source/Destination Type of Service*
*Total Packets*
*Total Bytes*
*Source Bytes*
*Label*

At the end of this phase, for each considered dataset we obtain one collection containing all the benign flows, and several collections of malicious flows generated by each botnet family included in each original dataset (namely, 5 collections for CTU, 1 for CICIDS and 5 for BOTNET). As a further benefit, the flows of the datasets are characterized by a uniform representation that is ready for the preprocessing phase.

### 2.2 Preprocessing

This phase performs some preliminary operations on the resulting flows after Data Normalization. The goal is to generate ready-to-use datasets for training state-of-the-art level botnet detectors. In this phase, we eliminate outliers and unwanted traffic, and we enrich the feature set with additional derived features leading the considered classifiers to achieve superior detection performance.

The first filtering operation removes all the non-TCP traffic included in CTU, CICIDS and BOTNET. As the botnet flows of the datasets are mainly based on modern IRC and HTTP protocols using TCP, this operation allows us to focus on a specific transport protocol, while maintaining most traffic. Moreover, we filter out the samples containing either NaN or unavailable values in one or more of their features.

Then, the samples are processed to compute the following derived features: *TotBytes, BytesPerPkt, PktsPerSec, RatioOutIn* (Table 6), when they are not present. As pointed in [4], the inclusion of these features can improve detection rate. Some samples come with null *Duration* and *InBytes*, which may result in infinite values for the *PktsPerSec* and *RatioOutIn*. Hence, we replace the infinite values with the maximum finite value of the correspondent feature in the considered dataset. For example, let us assume that a flow f has 0 *InBytes* and 300 *OutBytes*. To compute the *RatioOutIn* value, we should calculate the ratio between *OutBytes* and *InBytes*, but this operation would result in an infinite value. Thus, we replace this value with the maximum finite value in the considered dataset for *RatioOutIn*.

**Table 6:** Derived features.

Derived feature	Description	Type
*TotBytes*	Total number of Bytes in the communication. Sum of *OutBytes* and *InBytes* feature values.	Float
*BytesPerPkt*	Average number of bytes exchanged per packet. Ratio between *TotBytes* and *TotPkts*.	Float
*PktsPerSec*	Average number of packets exchanged per second. Ratio between *TotPkts* and *Duration*.	Float
*RatioOutIn*	Ratio between *OutBytes* and *InBytes*.	Float

We also remove *outlier* samples by considering only the flows in which the numerical feature values are below a threshold that is set according to the 95th-percentiles of the numerical feature values of the flows in the CTU dataset. We consider the CTU as a baseline because it contains the highest amount of samples and is a meaningful representation of a realistic scenario. The threshold values are reported below:

- Duration < 300 s (5 min)
- InBytes < 60,000
- OutBytes < 10,000
- TotPkts < 100
- BytesPerSec < 400,000
- PktsPerSec < 10,000

The application of these filters eliminates all the outliers while preserving the majority of malicious samples (over 90%).

We also observed that most malicious flows come from a narrow subset of network hosts, and the communications occur on a limited number of TCP ports. Training classifiers with similar features can cause overfitting problems and induce them to learn to distinguish malicious flows only on the basis of IP addresses and/or port numbers. For these reasons, we transform *Source* and *Destination IP Addresses* and *Port numbers* into the categorical features reported in Table 7. *IPSrcType* and *IPDstType* indicate whether or not the hosts involved in the communication belong to the enterprise internal network without expressively report the addresses. Similarly, *PortWellKnown, PortRegistered* and *PortPrivate* indicate to which category belongs the port number of the original sample. Thanks to these operations, we avoid training issues while maintaining information on the overall structure of the network.

**Table 7:** Categorical features.

Feature	Description	Type
*IPSrcType* / *IPDstType*	It indicates whether the *Source* / *Destination Ip Address* belongs to the internal network or not.	Binary (0 external, 1 internal).
*SrcPortWellKnown* / *DstPortWellKnown*	1 if the *Source* / *Destination Port* number is between 0 and 1023. 0 otherwise.	Binary (0 or 1).
*SrcPortRegistered* / *DstPortRegistered*	1 if the *Source* / *Destination Port* number is between 1024 and 49,151. 0 otherwise.	Binary (0 or 1).
*SrcPortPrivate* / *DstPortPrivate*	1 if the *Source* / *Destination Port* number is higher than 49,151. 0 otherwise.	Binary (0 or 1).

Finally, we perform a one-hot encoding operation to make remaining categorical features suitable to the neural networks of the DRL agents of the next phase. The final set of features for samples belonging to CTU, CICIDS and BOTNET are listed in Table 2 of the previous section.

At the end of the preprocessing phase we obtain ready-to-use samples to train state-of-the-art botnet detectors and DRL agents.

### 2.3 Automatic adversarial sample generation

This phase focuses on the generation of the adversarial samples that are contained in the DReLAB dataset. These samples are generated by using the malicious flows of the three considered sources (CTU, CICIDS, BOTNET) and applying small modifications to a subset of their features by means of DRL algorithms. This phase can be divided into three steps: proposal of botnet classifiers that achieve state-of-the-art detection performance; use of these classifiers as a basis to train DRL agents evading detection; letting the trained DRL agents generate the adversarial samples by applying the modifications learned in the previous step.

Experiments are performed on a machine based on Intel Core i7-7700HQ CPU (2.80 GHz x 4), 16 GB RAM, 1TB SSD, Nvidia Geforce GTX 1050M.

• We base our botnet detectors on two famous ML and DL algorithms: **Random Forest** (RF) and **Wide and Deep** (WnD). The **RF** algorithm consists of an ensemble learning method that uses multiple **Decision Trees** to yield its final classification. **WnD** is a deep learning technique proposed by Google that obtains good classifications results in other contexts [9]. Each detector is composed by an ensemble of classifiers, where each classifier is trained to identify one specific botnet family from legitimate traffic. Thus, we obtain 5 **RF** and 5 **WnD** classifiers for both the CTU and BOTNET datasets (one for each of the botnet variants in these datasets), 1 **RF** and 1 **WnD** classifiers for CICIDS. To reproduce a realistic enterprise network scenario, we add benign samples to the malicious collections in a 20:1 ratio as suggested by the best practice in literature [10]. The training set and the testing set use 80% and 20% of the samples, respectively. Table 8 and Table 9 report the parameters for **RF-** and **WnD-**based classifiers. To implement and train the classifiers we use the Scikit-learn framework [11] (version 0.21.2). The provided DReLAB dataset allows the implementation of classifiers with high detection rates, achieving Recall scores often superior to 0.95 (refer to the primary research paper [6] for more information).

**Table 8:** Parameters for RF classifiers.

Parameter	Value
Number of Estimators	100
Quality function	Gini
Minimum samples to split	2
Minimum samples to leaf	1
Features for best split	$\sqrt{N.\;Features}$

**Table 9:** Parameters for WnD classifiers.

Parameter	Value
Wide part - input neurons	256
Deep part - number of layers	5
Deep part - Neurons in each hidden layer	64 – 16 – 16 – 4 - 4
Activation	ReLU
Optimizer	Adam
Alpha	0.0001
• A DRL framework involves the cooperation between an agent and an environment. The agent learns to choose the best action among the pre-defined *Action Space* with a trial-and-error approach, while the environment analyzes this choice and provides a *Reward* to the agent that indicates the goodness of the chosen action. In order to produce adversarial samples that preserve their malicious functions, in defining our Action Space we consider a small subset of the available features: *Duration, InBytes, OutBytes* and *Total Packets*. Moreover, our *Action Space* includes only actions that modify the selected feature by small pre-fixed increments of at most two units (see Table 10). As an example, the agent can choose to increase the *Duration* by 1 or 2 s; similarly, it can add 1 or 2 packets to the *Total Packets* feature value or 1 or 2 bytes to *InBytes* and *Outbytes.* We remark that we use the **RF** detectors as target classifiers to evade because they achieve superior performance than the WnD detectors.

**Table 10:** Action space.

Modified feature	Perturbation
*Duration*	+1 or +2
*InBytes*	+1 or +2
*OutBytes*	+1 or +2
*TotPkts*	+1 or +2

The workflow for training a DRL agent to produce adversarial samples related to a botnet family b proceeds as follows (we refer to Figure 4 of the related research paper [6] for an illustration of the process). Each malicious flow of b, which we denote f_b_, is processed and modified individually. It is initially passed to the *state generator*, which transforms f_b_ in a suitable form to be submitted to the agent. Then, the agent analyzes the (transformed) f_b_ and choses one action from the pre-defined *Action Space* that, once applied to the sample, can evade detection. The application of the chosen action occurs in the *state generator* that avoids inconsistencies by updating the feature values of the corresponding derived features. For instance, an increase of *InBytes* causes a consequent increment of *BytesPerSecond*. The modified sample is then submitted to the classifier to test whether it evades or not detection. If the modified sample is misclassified, the process terminates and we obtain an adversarial sample. In this case, a positive *Reward* (+10 in our implementation) is given to the agent that modifies its internal weights to favor the action choice that has led to evasion. Otherwise, if the sample is detected as malicious, it is further modified until it is able to evade the classifier, or until a maximum number of attempts (*Qmax*) is reached.
We consider two DRL agents: **Double Deep Q-Network** (2DQN) [12] and **Deep Sarsa** [13] (**Sarsa**). We implemented our environments on OpenAI Gym^5^ that is a toolkit for reinforcement learning algorithms. Moreover, we used Keras-RL,^6^ a Python library that offers DRL agents algorithms, to implement the agents. All the **2DQN** and **Sarsa** agents share the same underlying neural network structure, which consists of three layers: the first contains as many neurons as the number of features as input; the second layer contains 16 neurons; the third layer contains one neuron for each action in the *Action Space.* We train two agents to generate adversarial samples that evades the **RF** classifier for each of the botnet families of the three datasets. Similarly to the botnet detectors, we obtain 5 **2DQN** and 5 **Sarsa** agents for evading the **RF** detectors of the CTU dataset, 1 **2DQN** and 1 **Sarsa** agents for CICIDS and 5 **2DQN** and 5 **Sarsa** agents for BOTNET. We provide the settings for both the DRL algorithms in Table 11.

**Table 11:** Settings of DRL agents during training.

Parameter	Description	Value
Qmax	Maximum number of modifications allowed before declaring failure	80
Reward	Reward in case of evasion	10
Policy	Type of policy for the training phase	Epsilon Greedy
Epsilon	Value of Epsilon for Epsilon Greedy Exploration	0.1
Replay buffer max size	Capacity of the experience replay buffer	50,000

• After having trained the DRL agents, we can now let them generate the adversarial samples that compose the proposed DReLAB dataset. To this purpose, we follow the same procedure as in the previous step but, as the agents are already trained, the environment does not emit any *Reward*. Let us explain the entire procedure by example. Suppose we want to obtain an adversarial sample from a malicious flow f of the botnet Neris from the CTU dataset, that evades the **WnD** detector by leveraging the **2DQN** agent.

The workflow proceeds as follows:

i f is submitted to the state generator to obtain its transformed version which is sent to the agent.
ii Now, the 2DQN agent has already learned which is the best action to choose to possibly obtain an evasion. Let us suppose that the chosen action is “Increase Duration + 2″.
iii The agent communicates this selection to the state generator, which adds 2 to the current Duration value of f. Then, the state generator also updates the derived PktsPerSec value to conform with the new Duration value. At this stage, the number of actions required for the evasion of f is increased by 1 (with 0 being its initial value).
iv The modified version of f (denoted as f’) is now a candidate for being an adversarial evasive sample. Thus, it is submitted to the WnD classifier trained on the Neris traffic from the CTU dataset and the classification is analyzed:
  ■ If the classifier still classifies the sample as malicious, then the process is restarted from point (i.) with the newly generated f’.
  ■ on the other hand, if f’ is classified as benign, then the process ends.

As shown in the related paper [6], the generated adversarial samples are able of evading not only the **RF** detector used for training the DRL agents, but also the **WnD** detector. In the dataset we include only those perturbed samples that are able of evading the detection with less than 80 actions. As an additional contribution, we also provide the number of actions required for the evasion in a dedicated feature to each of the adversarial sample of the DReLAB dataset.

## CRediT Author Statement

**Andrea Venturi, Giovanni Apruzzese:** Software, Data curation, Formal analysis, Writing – Original Draft, Writing Review & editing.

**Mauro Andreolini, Michele Colajanni, Mirco Marchetti:** Supervision, Validation, Writing – Review & Editing.

## Declaration of Competing Interest

All the authors declare that they have no known competing financial interests or personal relationships which have, or could be perceived to have, influenced the work reported in this article.

## Footnotes

1 https://openargus.org/

2 https://registry.opendata.aws/cse-cic-ids2018/

3 https://www.stratosphereips.org/blog/2015/7/17/new-dataset-ctu-13-extended-now-includes-pcap-files-of-normal-traffic

4 https://www.unb.ca/cic/datasets/botnet.html

5 https://gym.openai.com

6 https://github.com/keras-rl/keras-rl
